# Patterns of negative trauma laparotomy worldwide: a sub-analysis of a global dataset

**DOI:** 10.1007/s00068-026-03289-z

**Published:** 2026-08-18

**Authors:** William L. A. Laird, Michael F. Bath, Joachim Amoako, Jared Wohlgemut, Carlos M. Nuño-Guzmán, Monty Khajanchi, Brandon G. Smith, Laura Hobbs, Zane B. Perkins, Thomas G. Weiser, Timothy C. Hardcastle, Tom Bashford

**Affiliations:** 1 International Health Systems Group, Department of Engineering, University of Cambridge, Cambridge, UK; 2 University of Ghana Medical School, Accra, Ghana; 3 Department of Surgery, Korle Bu Teaching Hospital, Accra, Ghana; 4 Centre for Trauma Sciences, Blizard Institute, Queen Mary University of London, London, UK; 5 Queen Elizabeth Hospital Birmingham, University Hospitals Birmingham NHS Foundation Trust, Birmingham, UK; 6 Hospital Civil de Guadalajara Fray Antonio Alcalde, Guadalajara, Jalisco México; 7 Centro Universitario de Ciencias de la Salud, Universidad de Guadalajara, Guadalajara, Jalisco México; 8 Seth Gordhandas Sunderdas Medical College and King Edward Memorial Hospital, Mumbai, India; 9 Department of Anaesthesia, East & North Hertfordshire NHS Trust, Stevanage, UK; 10 NIHR Global Health Research Group on Acquired Brain and Spine Injury, Cambridge, UK; 11 Major Trauma Service, Royal London Hospital, Barts Health NHS Trust, London, UK; 12 Department of Surgery, Stanford University, Palo Alto, CA USA; 13 Trauma and Burns Unit, KwaZulu-Natal Department of Health, Inkosi Albert Luthuli Central Hospital, Durban, 4058 South Africa; 14 Department of Surgical Sciences, Nelson R Mandela School of Clinical Medicine (NRMSM), University of KwaZulu-Natal, Durban, 4000 South Africa; 15 Department of Anaesthesia, Cambridge University Hospitals NHS Foundation Trust, Cambridge, UK

**Keywords:** Trauma, Negative laparotomy, Global surgery, Morbidity

## Abstract

**Purpose:**

Trauma remains a major contributor to global disability. While the trauma laparotomy can be a life-saving procedure in abdominal injury, a select proportion are performed with no pathology identified intra-operatively, potentially exposing patients to unnecessary morbidity. We aimed to assess the global prevalence of negative laparotomy and identify any influencing factors at both the patient- and system-level.

**Methods:**

This was a secondary analysis of the GOAL-Trauma study, a multicentre prospective international observational study on trauma laparotomy patients, conducted from April to December 2024. We defined a negative laparotomy as where no intra-abdominal injuries were identified during the index operation. A multivariable logistic regression was performed to identify predictive factors for a negative laparotomy.

**Results:**

Of the 1769 patients included, 128 patients (7.2%) underwent a negative laparotomy. Regression analysis demonstrated that a penetrating mechanism of injury (OR 2.37, CI:1.53–3.69, *p* < 0.001) and a lower grade of surgeon (surgical registrar relative to consultant - OR 2.91, CI:1.90–4.47, *p* < 0.001) were independent predictors for negative laparotomy. Neither human development index nor hospital resource level impacted negative laparotomy rates.

**Conclusion:**

One in every fourteen trauma laparotomies performed in our global cohort was negative, with injury type and surgeon experience having a significant deterministic role. As trauma systems develop globally, recognition in the value of both training and a structured assessment pathway is paramount. The rate of negative trauma laparotomy, either institutionally or regionally, may act as a useful benchmark of trauma system performance and warrants further exploration.

**Supplementary Information:**

The online version contains supplementary material available at 10.1007/s00068-026-03289-z.

## Introduction

Trauma is a leading cause of mortality globally, accounting for approximately 4.4 million deaths annually [1]. Severe injury can also result in significant morbidity and is responsible for an estimated 280 million disability-adjusted life years (DALYs) per year [2], equating to around 11% of the annual total global burden of disease. This is further compounded by trauma disproportionately affecting younger people of working age [3], resulting in substantial impacts on economic output [4, 5]. Consequently, a priority in the optimisation of trauma care must be to reduce not just mortality, but the associated morbidity and disability.

Abdominal injury is present in 10% of trauma patients [6] and for those with severe injury, the trauma laparotomy can be a life-saving intervention, both in terms of diagnosis and management. Appropriate clinical decision-making in trauma requires a combination of clinical history and examination, corroborated by suitable investigations in a time-critical manner [7]. Despite this, trauma patients will occasionally undergo a laparotomy in which no pathology can be identified: a “negative” laparotomy. Whilst a select number of such cases can be perceived as an inevitability within the trauma triage process, such a procedure still poses risks, including those from emergency general anaesthesia, wound complications, infection, and delayed recovery, not to mention the lifelong risk of post-surgery adhesive obstruction, and arguably with no immediate reciprocal benefit either. Furthermore, the human, material, and opportunity costs incurred by the institution, the patient, and their relatives from such a situation will inherently be sizeable. Whilst a negative laparotomy may be seen an inevitable part of trauma care, especially in certain high-risk patient groups as an investigative means to exclude injury, reducing their occurrence should regardless be seen as a priority to improve trauma patient outcomes.

Decisions and outcomes in trauma care are shaped not only by patient factors, but also by interconnected system-level factors that affect the patient pathway [8]. To understand and therefore reduce rates of negative trauma laparotomy, these system-level factors must be incorporated into any analysis. The Global Outcomes After Laparotomy for Trauma (GOAL-Trauma) study was an international multicentre prospective observational cohort study of patients undergoing trauma laparotomy [9]; using this dataset, this secondary analysis aimed to identify both patient- and system-level factors that may be associated with negative trauma laparotomies, in order to inform future targeted interventions to reduce this burden of unnecessary morbidity and cost.

## Methods

### Study design

This study is a secondary analysis of the GOAL-Trauma dataset. The GOAL-Trauma study was a prospective international multicentre observational cohort study, conducted from April to December 2024. The study has been reported has been reported according to the STrengthening the Reporting of OBservational studies in Epidemiology (STROBE) guidelines [10]. The study hypothesis was that both patient- and system-level factors would influence core post-operative outcome measures.

The GOAL-Trauma study methodology has been previously described in detail [11]. In brief, recruitment of hospitals was through open invitation, with potential collaborators contacted through a purposive snowballing technique, using pre-existing research networks, personnel contacts, and social media. The study recruited 187 centres across 51 countries, representing all six inhabited continents (Supplementary Information). Deidentified data were collected locally by collaborators at each centre and uploaded centrally onto a secure web-based system, REDCap cloud [12, 13], hosted by the University of Cambridge. Collaborators were given REDCap project server login details, allowing secure data entry and storage; each recruiting centre appointed a lead investigator (termed the ‘local lead’), who was invited to complete an online structured site survey regarding trauma care at their institution.

Procedure type was recorded by the collaborators following the initial operation. A negative laparotomy was defined as a procedure where no intra-abdominal injuries were identified during the index operation, whereas a non-therapeutic laparotomy was defined as a laparotomy where intra-abdominal injuries were identified however did not require any operative intervention. Injury severity was calculated using Abbreviated Injury Scale (AIS) scores for each body region, with the overall degree of injury quantified through the summative Injury Severity Score (ISS) [14]. Co-morbidity was quantified through the American Society of Anaesthesiologists (ASA) score. The Adapted Clavien–Dindo in Trauma (ACDiT) score was used as a measure of post-operative complications [15]. Contributing hospitals were stratified by their national Human Development Index (HDI) [16]; HDI tertiles were calculated by dividing all countries in the world into lower, middle, and upper tertiles based on their respective HDI ranking, with each country in the GOAL-Trauma study then assigned into their respective tertile.

The study received ethical approval from the Cambridge Psychology Research Ethics Committee (PRE.2023.119) and was registered to ClinicalTrials.gov (NCT06180668, registration date 22nd December 2023), with the study sponsored by the University of Cambridge (Cambridge, UK). Appropriate local approval was obtained by each contributing centre before enrolment into the study, according to respective local regulations. In some participating hospitals, informed patient consent was taken, whereas in other hospitals this was deemed not necessary, at the discretion of the local team. The conduct of the GOAL-Trauma study was overseen by an international steering committee consisting of expert clinical academic representatives across multiple economic and geographical settings.

### Statistical analysis

Descriptive statistics were calculated as mean and standard deviation, median and interquartile range, or number and percentage, where appropriate. Differences in outcome metrics across HDI tertiles were assessed with the Kruskal-Wallis test or χ² test, where appropriate.

Determining factors that influenced the likelihood of a negative laparotomy were assessed through multivariable logistic regression. Initial selection of covariates was guided via a directed acyclic graph (DAG), derived through a combination of literature review and expert consensus from the study’s steering committee, to ensure the model accounted appropriately for all pathways and factors between the exposure and the outcome. The model was subject to complete case restriction to participants with non-missing values for the outcome and all prespecified covariates included in that model. A hospital resource availability score was derived from ordinal resource availability measures derived from the site survey (intensive care, computed tomography (CT) scan, pathology services, and blood products), producing a one-factor latent score using polychoric correlations and maximum-likelihood factor analysis with regression scoring, with Yeo-Johnson transformation. Due to a significant number of hospitals contributing very small number of patients, a hierarchical clustering model based on hospital was deemed not feasible.

All analyses were conducted on R (version 4.5.2), using the ‘readxl’, ‘dplyr’, ‘stringr’, ‘psych’, ‘bestNormalize’ and ‘scales’ packages. Effect estimates were reported as odds ratios (ORs) with 95% confidence intervals (CIs), with p-values < 0.05 considered statistically significant.

### Role of the funding source

The funders of the study had no role in study design, data collection or analysis, interpretation of the data, or in the writing of the report.

## Results

### Participant characteristics

As has been previously reported, the GOAL-Trauma study recruited 1769 patients, comprising 563 patients (31.8%) from the lower HDI tertile, 714 patients (40.4%) from the middle HDI tertile, and 492 patients (27.8%) from the upper HDI tertile. The median age was 30 years (IQR 23–43 years) and the majority of patients were male (1512 of 1769 patients, 85.5%). Similar rates of blunt versus penetrating mechanisms for trauma were observed overall (46.0% versus 54.0%, respectively), yet higher rates of penetrating injury were seen in the lower HDI tertile (337 of 563 patients, 59.9%, *p* < 0.0001) relative to the middle and upper HDI tertiles.

The most common injuries sustained were small bowel injuries (607 patients, 23.2%), followed by hepatobiliary (467 patients, 17.8%) and splenic injuries (419 patients, 16.0%) (Table 1). Overall, 128 patients (of 1769 patients, 7.2%) were recorded as having a negative laparotomy, while 76 patients (of 1769 patients, 4.3%) were recorded as having a non-therapeutic laparotomy; of the non-therapeutic laparotomy patients, the most common injuries were liver injuries (21 patients, 27.6%), followed by retroperitoneal haemorrhage (13 patients, 17.1%) and splenic injuries (10 patients, 13.2%).

**Table 1:** Prevalence of injuries sustained, grouped by region; n(%)

Injury Group	Total	HDI Tertile		
		Lower	Middle	Upper
Small Bowel	607 (23.2%)	199 (26.1%)	239 (22.4%)	169 (21.5%)
Hepato-Pancreato-Biliary	467 (17.8%)	133 (17.5%)	199 (18.6%)	135 (17.2%)
Spleen	419 (16.0%)	106 (13.9%)	167 (15.6%)	146 (18.6%)
Colorectal	389 (14.9%)	113 (14.8%)	155 (14.5%)	121 (15.4%)
Gastric (including Duodenal)	244 (9.3%)	83 (10.9%)	104 (9.7%)	57 (7.3%)
Urological	204 (7.8%)	61 (8.0%)	91 (8.5%)	52 (6.6%)
Vascular	111 (4.2%)	16 (2.1%)	53 (5.0%)	42 (5.3%)
Gynaecological	4 (0.2%)	1 (0.1%)	1 (0.1%)	2 (0.3%)
Other	172 (6.6%)	50 (6.6%)	60 (5.6%)	62 (7.9%)

### Negative laparotomy cohort

There were 128 patients in the negative laparotomy cohort and 1641 patients in the non-negative laparotomy cohort (Table 2). The highest proportion of negative laparotomy occurred in the lower HDI tertile (55 of 563 patients, 9.8%, *p* = 0.016), compared to both the middle HDI tertile (46 of 714 patients, 6.4%) and the upper HDI tertile (27 out of 492 patients, 5.5%). The median age was 30 years and the majority (109 of 128 patients, 85.2%) were male.

**Table 2:** Characteristics between the negative laparotomy and non-negative laparotomy cohorts; values are n (%), unless otherwise indicated

	**Negative Laparotomy** (***n*** **= 128**)	**Non-Negative Laparotomy** (***n*** **= 1641**)	***p*** ** value**
**HDI Tertile **			
- Lower - Middle - Upper	55 (43.0%) 46 (35.9%) 27 (21.1%)	508 (31.0%) 668 (40.7%) 465 (28.3%)	0.016
**Age (years)**; median (IQR)	30 (22–40)	31 (23–43)	0.409
**Sex ratio**,** M : F**	109 : 19	1403 : 238	0.916
**Mechanism of Injury**			
- Penetrating - Blunt	95 (74.2) 33 (25.8)	860 (52.4) 781 (47.6)	< 0.001
**Pre-Operative CT scan**	62 (48.4%)	838 (51.1%)	0.567
**Systolic Blood Pressure (mmHg)**; median (IQR)			
- On arrival - At induction	110 (100–130) 115 (109–129)	110 (93–125) 110 (100–127)	0.017 0.056
**Surgeon Grade**			
- Consultant / Attending - Surgical Registrar / Resident - Non-General Surgeon Resident - Medically Qualified Non-Surgeon	62 (48.8%) 65 (51.2%) 0 (0%) 0 (0%)	1244 (75.8%) 385 (23.5%) 7 (0.4%) 5 (0.3%)	< 0.001
**ASA Grade**			
1 2 3 4 5	45 (35.2) 44 (34.3) 25 (19.5) 5 (3.9) 9 (7.0)	629 (38.3) 402 (24.5) 271 (16.5) 206 (12.6) 133 (8.1)	0.010
**Injury Severity Score**; median (IQR)	0 (0–2)	12 (9–22)	< 0.001
**Post-operative death**	10 (7.8)	185 (11.3)	0.228

The most common mechanism of injury in the negative laparotomy cohort was a penetrating injury (95 of 128 patients, 74.2%, *p* < 0.001), substantially higher than in the non-negative laparotomy group (860 of 1641 patients, 52.4%). Those with a blunt injury were more likely to have a Consultant / Attending surgeon (23 of 33 patients, 69.7%, *p* = 0.005), compared to patients with a penetrating injury (39 of 95 patients, 41.1%). Pre-operative CT imaging was performed in approximately half of all negative cases (62 of 128 patients, 48.4%), similar to the non-negative laparotomy group (838 of 1641 patients, 51.1%). Surgical registrars / residents were the most common surgeon grade present in the negative laparotomy group (65 of 128 patients, 51.2%, *p* < 0.001), compared to the non-negative laparotomy group, where the most common surgeon grade was consultant / attending (1244 of 1641 patients, 75.8%). The majority of cases underwent a primary closure at the index procedure (126 of 128 patients, 98.4%).

**Fig. 1:**
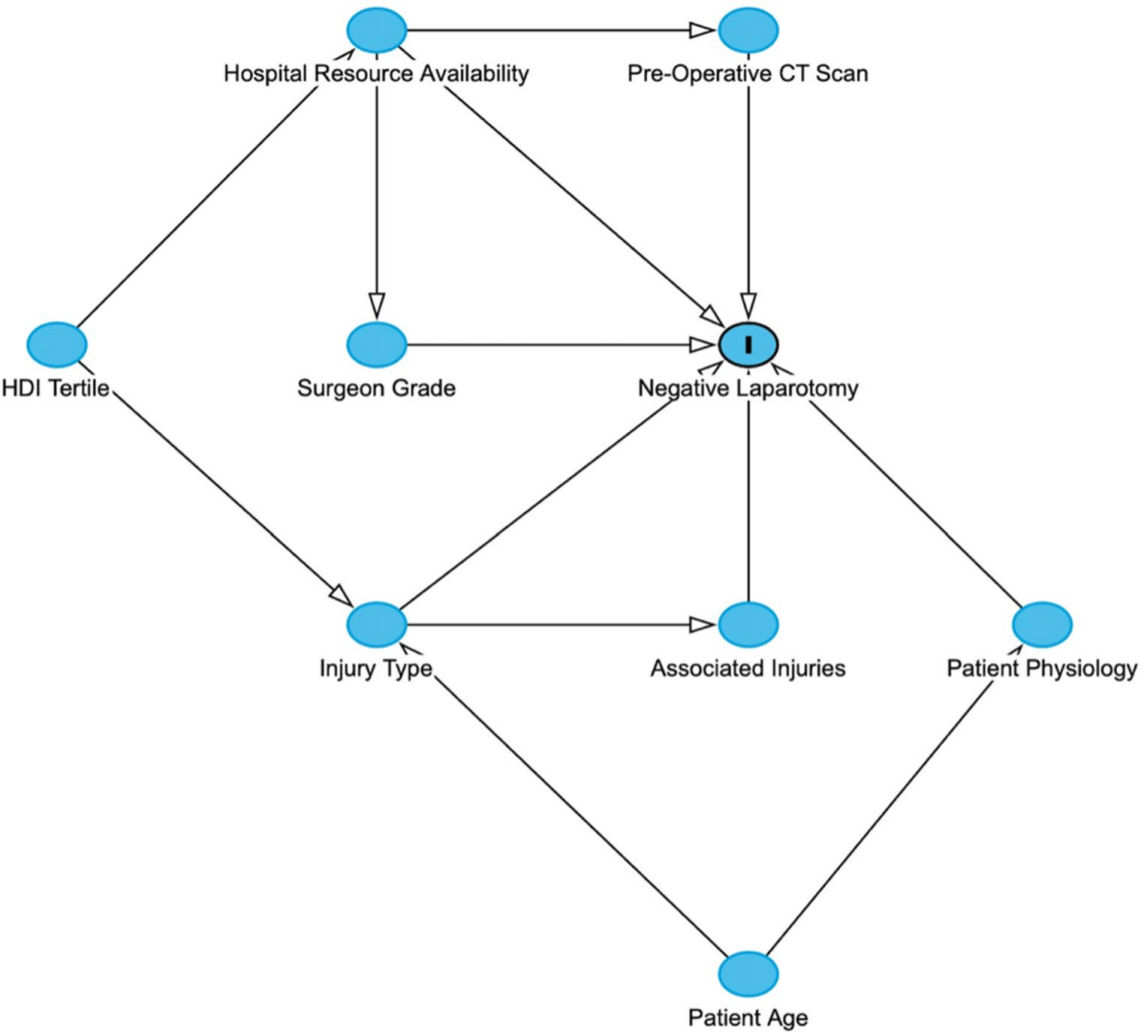
Directed acyclic graph demonstrating factors influencing risk of negative laparotomy

**Fig. 2:**
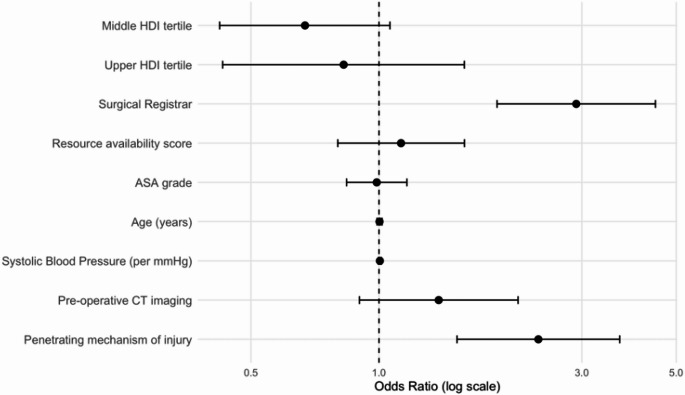
Forest plot demonstrating Odds Ratio and 95% confidence intervals for factors influencing risk of negative laparotomy

Following the selection of covariates based on the DAG (Fig. 1) and subsequent exclusion of those with incomplete data, 1765 patients (99.8%) were included in the model. Following adjustment through multivariable logistic regression, two significant factors were identified as predictors for negative laparotomy (Fig. 2): penetrating mechanism (OR 2.37, CI:1.53–3.69, *p* < 0.001) and grade of surgeon (surgical registrar relative to consultant - OR 2.91, CI:1.90–4.47, *p* < 0.001). Pre-operative CT imaging (OR 1.38, CI: 0.90–2.12, *p* = 0.140) showed a trend towards increased risk, however this was not significant, whilst neither HDI tertile nor resource availability showed any impact on the likelihood of negative laparotomy.

Of the 128 negative laparotomy patients, ten patients (7.8%) died in hospital within 30 days. Among those who died, six were from the lower HDI tertile, two were from the middle HDI tertile, and two were from the upper HDI tertile. The majority of the patients who died had sustained a blunt mechanism of injury (eight of ten patients, 80%) and had a median systolic blood pressure at induction of 90mmHg (IQR 61–100 mmHg), compared to 119mmHg (IQR 110–130mmHg) in those who survived. The majority of these patients had an ASA grade of 3 or higher (six of ten patients, 60%), compared to around a quarter in those who survived (33 of 118 patients, 27.9%).

Post-operative organ support was required in 21 patients (of 128 patients, 16.4%). A relook procedure was required in four patents (of 128 patients, 3.1%), of which half of these were unplanned. Of the 118 patients who survived, an ACDiT score was recorded in 113 patients, of which 11 (9.7%) had a score of ≥ 3 (suggestive of a severe complication).

## Discussion

With traumatic injuries resulting in substantial and increasing proportions of disability worldwide, reducing the rate of negative laparotomy in trauma care should be viewed as an opportunity to limit both patient morbidity and resource allocation, and therefore a global priority. Indeed, within our international cohort, the rates of mortality, post-operative complication, and morbidity for those who received a negative laparotomy were substantial. Globally, we observed a negative laparotomy rate of 7.2%, equating to one in fourteen procedures that could be defined as potentially avoidable. While a penetrating mechanism of injury and more junior grade of surgeon were found to significantly increase the likelihood of a negative laparotomy, factors conventionally associated with best practice, such as pre-operative CT imaging, showed a trend towards an increase risk as well.

Understanding the influencing factors for a negative laparotomy and how they interact is key if their rates are to be reduced on the global stage. Negative laparotomies should not just be viewed as a procedure where no injuries are identified, as indeed a select proportion of negative findings can be expected for any triaging service, but instead that a negative laparotomy is an intervention that exposes the patient to a significant risk of morbidity and mortality. In this work, we found one in six patients required post-operative organ support and one in ten patients developed a severe post-operative complication following a trauma laparotomy. Previous work in this area has demonstrated the complexity inherently present within trauma care [17], therefore identifying a single modifiable factor that alone could reduce the rate of negative laparotomy is unlikely. To understand the causal associations among the included variables, we used a DAG-informed regression analysis that included factors at both the patient- and system-level from across the trauma care pathway, which led to key clinically-relevant findings to be identified.

Historically, peritoneal breach in penetrating abdominal trauma was considered an indication for mandatory laparotomy; although this approach has been superseded by selective non-operative management in many settings, elements of this paradigm may persist in clinical decision-making, with some clinical protocols still advising that any penetrating trauma warrants exploratory laparotomy [18]. As a result, it is unsurprising that in our analysis a penetrating mechanism was shown to increase the risk of a negative laparotomy. However, we also demonstrated that a more junior grade of surgeon was associated with an increased risk. While there are selection biases in this conclusion, such as patients with more serious suspected injuries warranting a more senior surgeon to operate [19], or the grade of surgeon making the actual decision to operate not being recorded in the analysis, this could still represent a risk-averse behaviour in the face of uncertainty or a reduced diagnostic experience in the more junior surgeons. Indeed, it is a recurring trend in the literature that clinicians with more experience are more tolerant of uncertainty and are less risk-averse in their clinical decision-making, thereby reducing their reliance on diagnostic tests [20, 21]. Regardless, these findings emphasise the importance of appropriate dedicated training pathways in trauma care and senior support in decision-making.

We further observed a non-significant trend towards those who underwent a pre-operative CT scan being more likely to have a negative laparotomy performed. While CT scans are a critical element of abdominal trauma investigations in many settings [18], the selection of borderline clinical cases for CT evaluation and over-interpretation of equivocal radiological findings can lead to an increased likelihood of a negative laparotomy being performed. Neither the HDI tertile nor the resource availability score of a hospital was shown to affect the likelihood of a negative laparotomy, suggesting that staff training and experience take precedence over any individual resource availability. It is further worth noting that whilst we have assessed the impact CT imaging has as a diagnostic modality, due to the study inclusion criteria, the role and impact of laparoscopy as a diagnostic tool was not assessed; use of laparoscopy may mitigate the risk of an unnecessary laparotomy in selected trauma patients with a penetrating mechanism, particularly where diagnostic uncertainty drives operative decision-making.

The trauma laparotomy is a bellwether procedure of global trauma care [22], a means by which trauma systems can be benchmarked across settings. Within the broader surgical literature, caesarean section (CS) rates have been suggested as a marker of surgical system function [23], both as a proportion of live births and as a proportion of general surgical cases [24]. Low CS rates relative to live births suggest critical system deficits, while high CS rates relative to all cases also suggest a weaker system with over-reliance on surgical intervention and suboptimal patient selection. Similarly, a negative laparotomy should not be viewed solely as a complication, but as an expected consequence of decision-making under time-pressure and diagnostic uncertainty, and a degree of negative exploration may therefore be unavoidable in trauma care. However, very low rates may reflect a high threshold for operative intervention, with the potential risk of missed or delayed treatment for time critical injuries, whereas higher rates may indicate avoidable morbidity from unnecessary surgery. In the context of previously published national data [25, 26], our observed negative laparotomy rate of 7% may reflect an appropriate balance between timely intervention and diagnostic uncertainty. Indeed, using the negative laparotomy rate as a measure of trauma system function is unique as it captures both patient and system factors. Further research is required to understand the extent to which this rate is context-dependent, particularly with respect to injury epidemiology [27], however it has the potential to provide a metric by which clinicians may benchmark their trauma system against local and global standards.

This study uses one of the largest global datasets on trauma laparotomy to date, along with a robust analytical approach. However, it does come with limitations. The reporting of negative laparotomy was self-reported by the study collaborators, with a proportion of those patients ultimately going on to die of their injuries; this mortality may represent extra-abdominal injury or subsequent complications (such as thromboembolism), but we cannot exclude the fact that some cases may have had a missed pathology at the indexed procedure. A high proportion of the included hospitals were tertiary urban centres, limiting the applicability of our findings to rural areas, a common problem with global observational studies on trauma care [28]. The dataset also has limited granularity in key decision-making variables, especially regarding the specific extent to which clinicians are involved in pre-operative decision making, as well as in morbidity and disability metrics, with no post-discharge functional assessments included. Similarly, whilst the use of the ACDiT score provided a quantifiable comparator for trauma-related complications, we are unable to state the exact nature of any complication, whether from the trauma laparotomy or any other related injuries. As a secondary analysis, there remains an increased risk of type 2 error, and we have been cautious not to overstate our study findings. Finally, the observational nature of the study introduces a risk of selection bias and unmeasured confounders, despite our use of DAGs in an attempt to address this.

## Conclusion

A negative trauma laparotomy incurs both avoidable morbidity and resource cost without therapeutic benefit. Our findings suggest that surgeon experience and a presentation of penetrating trauma play a significant role in the likelihood of a negative trauma laparotomy, with the use of CT imaging possibly increasing this likelihood further, albeit without statistical significance for this finding. In contrast, we found no association with HDI tertile or resource availability, suggesting that negative laparotomies are not simply a function of either well-resourced or under-resourced trauma systems. Structured assessment pathways, along with early senior involvement in cases of equivocal abdominal trauma, may be key to avoiding this morbidity. We propose that the rate of negative trauma laparotomy may represent a novel indicator of trauma system functionality, however requires much further exploration before being formally recommended.

## Supplementary Information

Below is the link to the electronic supplementary material.

Supplementary Material 1

## Data Availability

No datasets were generated or analysed during the current study.

## References

[1] World Health Organization. Injuries and violence: the facts. Geneva; 2014.

[2] Murray CJL, Vos T, Lozano R, Naghavi M, Flaxman AD, Michaud C, et al. Disability-adjusted life years (DALYs) for 291 diseases and injuries in 21 regions, 1990–2010: a systematic analysis for the Global Burden of Disease Study 2010. Lancet. 2012;380(9859):2197–223. 10.1016/S0140-6736(12)61689-4. 23245608 10.1016/S0140-6736(12)61689-4

[3] Mathers CD, Loncar D. Projections of global mortality and burden of disease from 2002 to 2030. PLoS Med. 2006;3(11):2011–30. 10.1371/journal.pmed.0030442 . PubMed PMID: 17132052. 10.1371/journal.pmed.0030442 PMC1664601 17132052

[4] Vos T, Lim SS, Abbafati C, Abbas KM, Abbasi M, Abbasifard M, et al. Global burden of 369 diseases and injuries in 204 countries and territories, 1990–2019: a systematic analysis for the Global Burden of Disease Study 2019. Lancet. 2020;396(10258):1204–22. 10.1016/S0140-6736(20)30925-9. 33069326 10.1016/S0140-6736(20)30925-9 PMC7567026

[5] Christie SA, Dickson D, Mbeboh SN, Embolo FN, Chendjou W, Wepngong E, et al. Association of Health Care Use and Economic Outcomes After Injury in Cameroon. JAMA Netw Open. 2020;3(5):e205171. 10.1001/jamanetworkopen.2020.5171. 32427321 10.1001/jamanetworkopen.2020.5171 PMC7237963

[6] Wiik Larsen J, Søreide K, Søreide JA, Tjosevik K, Kvaløy JT, Thorsen K. Epidemiology of abdominal trauma: An age- and sex-adjusted incidence analysis with mortality patterns. Injury. 2022;53(10):3130–8. 10.1016/j.injury.2022.06.020. 35786488 10.1016/j.injury.2022.06.020

[7] Como JJ, Bokhari F, Chiu WC, Duane TM, Holevar MR, Tandoh MA, et al. Practice Management Guidelines for Selective Nonoperative Management of Penetrating Abdominal Trauma. J Trauma: Injury Infect Crit Care. 2010;68(3):721–33. 10.1097/TA.0b013e3181cf7d07. 10.1097/TA.0b013e3181cf7d07 20220426

[8] Bashford T, Clarkson PJ, Menon DK, Hutchinson PJA. Unpicking the Gordian knot: a systems approach to traumatic brain injury care in low-income and middle-income countries. BMJ Glob Health. 2018;3(2):e000768. 10.1136/bmjgh-2018-000768 . PubMed PMID: 29607105. 29607105 10.1136/bmjgh-2018-000768 PMC5873538

[9] Bath MF, Amoako J, Kohler K, Hashi AS, Zhang Z, Baderhabusha DU, et al. Global variation in patient factors, interventions, and postoperative outcomes for those undergoing trauma laparotomy: an international, prospective, observational cohort study. Lancet Glob Health. 2025;13(11):e1837–48. 10.1016/S2214-109X(25)00303-1. 40972623 10.1016/S2214-109X(25)00303-1

[10] von Elm E, Altman DG, Egger M, Pocock SJ, Gøtzsche PC, Vandenbroucke JP. The Strengthening the Reporting of Observational Studies in Epidemiology (STROBE) statement: guidelines for reporting observational studies. Lancet. 2007. 10.1016/S0140-6736. (07)61602-X PubMed PMID: 18064739. 10.1016/S0140-6736(07)61602-X 18064739

[11] Bath MF, Kohler K, Hobbs L, Smith BG, Clark DJ, Kwizera A, et al. Evaluating patient factors, operative management and postoperative outcomes in trauma laparotomy patients worldwide: a protocol for a global observational multicentre trauma study. BMJ Open. 2024;14(4):e083135. 10.1136/bmjopen-2023-083135 . PubMed PMID: 38580358. 38580358 10.1136/bmjopen-2023-083135 PMC11002395

[12] Harris PA, Taylor R, Minor BL, Elliott V, Fernandez M, O’Neal L, et al. The REDCap consortium: Building an international community of software platform partners. J Biomed Inf. 2019;95:103208. 10.1016/j.jbi.2019.103208. 10.1016/j.jbi.2019.103208 PMC7254481 31078660

[13] Harris PA, Taylor R, Thielke R, Payne J, Gonzalez N, Conde JG. Research electronic data capture (REDCap)—A metadata-driven methodology and workflow process for providing translational research informatics support. J Biomed Inf. 2009;42(2):377–81. 10.1016/j.jbi.2008.08.010. 10.1016/j.jbi.2008.08.010 PMC2700030 18929686

[14] Gennarelli T, Wodzin E. Abbreviated injury scale (c) 2005 update 2008 [Internet]. Chicago, IL; 2016 [cited 2023 Jul 12]. Available from: https://www.aaam.org/abbreviated-injury-scale-ais/about-ais/

[15] Naumann DN, Vincent LE, Pearson N, Beaven A, Smith IM, Smith K, et al. An adapted Clavien-Dindo scoring system in trauma as a clinically meaningful nonmortality endpoint. J Trauma Acute Care Surg. 2017;83(2):241–8. 10. 1097/TA.0000000000001517 PubMed PMID: 28731937. 28731937 10.1097/TA.0000000000001517 PMC5527750

[16] United Nations Development Programme. Human Development Data [Internet]. 2025. Available from: https://hdr.undp.org/data-center

[17] Amoako J, Bath MF, Wordui T, Ametefe EK, Komashie A, Kohler K, et al. Visualisation of a complex healthcare system: mapping vascular and trauma surgery services in Ghana. BMC Health Serv Res. 2026;26(1):241. 10.1186/s12913-025-13982-1 . PubMed PMID: 41559754. 41559754 10.1186/s12913-025-13982-1 PMC12903557

[18] Smyth L, Bendinelli C, Lee N, Reeds MG, Loh EJ, Amico F, et al. WSES guidelines on blunt and penetrating bowel injury: diagnosis, investigations, and treatment. World J Emerg Surg. 2022;17(1):13. 10.1186/s13017-022-00418-y. 35246190 10.1186/s13017-022-00418-y PMC8896237

[19] Marsden M, Carden R, Navaratne L, Smith IM, Penn-Barwell JG, Kraven LM, et al. Outcomes following trauma laparotomy for hypotensive trauma patients: A UK military and civilian perspective. J Trauma Acute Care Surg. 2018;85(3):620–5. 10. 1097/TA.0000000000001988 PubMed PMID: 29847536. 29847536 10.1097/TA.0000000000001988

[20] Valentine K, Leavitt L, Sepucha KR, Atlas SJ, Simmons L, Siegel L, et al. Uncertainty tolerance among primary care physicians: Relationship to shared decision making-related perceptions, practices, and physician characteristics. Patient Educ Couns. 2024;123:108232. 10.1016/j.pec.2024.108232. 38458091 10.1016/j.pec.2024.108232 PMC10997439

[21] Lawton R, Robinson O, Harrison R, Mason S, Conner M, Wilson B. Are more experienced clinicians better able to tolerate uncertainty and manage risks? A vignette study of doctors in three NHS emergency departments in England. BMJ Qual Saf. 2019;28(5):382–8. 10.1136/bmjqs-2018-008390 . PubMed PMID: 30728187. 30728187 10.1136/bmjqs-2018-008390 PMC6560462

[22] Bath MF, Amoako J, Edmiston T, Ratnayake AS, Kabatoro D, Bagaria D, et al. Defining the bellwether procedures and processes for global trauma care: an international Delphi study. BMJ Glob Health. 2026;11(2). 10.1136/bmjgh-2025-020909. PubMed PMID: 41720581. 10.1136/bmjgh-2025-020909 PMC12927294 41720581

[23] Betran AP, Torloni MR, Zhang J, Ye J, Mikolajczyk R, Deneux-Tharaux C, et al. What is the optimal rate of caesarean section at population level? A systematic review of ecologic studies. Reprod Health. 2015;12:57. 10.1186/s12978-015-0043-6 . PubMed PMID: 26093498. 26093498 10.1186/s12978-015-0043-6 PMC4496821

[24] Petroze RT, Mehtsun W, Nzayisenga A, Ntakiyiruta G, Sawyer RG, Forrest Calland J. Ratio of Cesarean Sections to Total Procedures as a Marker of District Hospital Trauma Capacity. World J Surg. 2012;36(9):2074–9. 10.1007/s00268-012-1629-6. 22532310 10.1007/s00268-012-1629-6 PMC3460261

[25] Schnüriger B, Lam L, Inaba K, Kobayashi L, Barbarino R, Demetriades D. Negative laparotomy in trauma: are we getting better? Am Surg. 2012;78(11):1219–23. PubMed PMID: 23089438. 23089438

[26] Atkins K, Schneider A, Charles A. Negative laparotomy rates and outcomes following blunt traumatic injury in the United States. Injury. 2023;54(8):110894. 10.1016/j.injury.2023.110894 . PubMed PMID: 37330406. 37330406 10.1016/j.injury.2023.110894 PMC10526723

[27] Whittaker G, Norton J, Densley J, Bew D. Epidemiology of penetrating injuries in the United Kingdom: A systematic review. Int J Surg. 2017;41:65–9. 10.1016/j.ijsu.2017.03.051. 28343028 10.1016/j.ijsu.2017.03.051

[28] Obermeyer Z, Abujaber S, Makar M, Stoll S, Kayden SR, Wallis LA, et al. Emergency care in 59 low- and middle-income countries: a systematic review. Bull World Health Organ. 2015;93(8):577–G586. 10.2471/BLT.14.148338 . PubMed PMID: 26478615. 26478615 10.2471/BLT.14.148338 PMC4581659

